# Supplementary material for: Development of a computer-aided design software for the quantitative evaluation of aesthetic damage
Source: PLoS One. 2019 Dec 18;14(12):e0226322. doi: 10.1371/journal.pone.0226322 (PMC6919621; doi:10.1371/journal.pone.0226322)
Supplement: S1 Code site — 1. (ZIP) [file pone.0226322.s001.zip › [EESC_jr][Projeto]Relatorio_07_21/reports/templates_zip/gentelella-master/vendors/Flot/examples/series-errorbars/index.html]

Flot Examples: Error Bars


## Error Bars

With the errorbars plugin you can plot error bars to show standard deviation and other useful statistical properties.

Copyright © 2007 - 2014 IOLA and Ole Laursen
